# A case of fatal Type I congenital disorders of glycosylation (CDG I) associated with low dehydrodolichol diphosphate synthase (DHDDS) activity

**DOI:** 10.1186/s13023-016-0468-1

**Published:** 2016-06-24

**Authors:** S. Sabry, S. Vuillaumier-Barrot, E. Mintet, M. Fasseu, V. Valayannopoulos, D. Héron, N. Dorison, C. Mignot, N. Seta, I. Chantret, T. Dupré, S. E. H. Moore

**Affiliations:** INSERM U1149, Faculté de Médecine Xavier Bichat, 16 rue Henri Huchard, Paris, France; Université Denis Diderot, Paris 7, Paris, France; Université Pierre et Marie Curie, Paris 6, Paris, France; Biochemical Genetics Department, Human Genetics Division, National Research Center NRC, Cairo, Egypt; AP-HP, Hôpital Bichat-Claude Bernard, Biochimie, Paris, France; Département de Pédiatrie, AP-HP, Hôpital Necker-Enfants Malades, Paris, France; Département de Génétique & Centre de Référence Déficiences Intellectuelles de Causes Rares, Hôpital Pitié Salpétrière, Paris, France; Groupe de Recherche Clinique « Déficience Intellectuelle et Autisme » UPMC, Paris, France; Neuropédiatrie, Hôpital Trousseau, Paris, France; Université Paris Descartes, Paris, France

**Keywords:** Protein N-glycosylation, Dolichol linked oligosaccharide, Retinitis pigmentosa, Endoplasmic reticulum

## Abstract

**Background:**

Type I congenital disorders of glycosylation (CDG-I) are mostly complex multisystemic diseases associated with hypoglycosylated serum glycoproteins. A subgroup harbour mutations in genes necessary for the biosynthesis of the dolichol-linked oligosaccharide (DLO) precursor that is essential for protein N-glycosylation. Here, our objective was to identify the molecular origins of disease in such a CDG-Ix patient presenting with axial hypotonia, peripheral hypertonia, enlarged liver, micropenis, cryptorchidism and sensorineural deafness associated with hypo glycosylated serum glycoproteins.

**Results:**

Targeted sequencing of DNA revealed a splice site mutation in intron 5 and a non-sense mutation in exon 4 of the dehydrodolichol diphosphate synthase gene (DHDDS). Skin biopsy fibroblasts derived from the patient revealed ~20 % residual DHDDS mRNA, ~35 % residual DHDDS activity, reduced dolichol-phosphate, truncated DLO and *N*-glycans, and an increased ratio of [2-^3^H]mannose labeled glycoprotein to [2-^3^H]mannose labeled DLO. Predicted truncated DHDDS transcripts did not complement *rer2*-deficient yeast. SiRNA-mediated down-regulation of DHDDS in human hepatocellular carcinoma HepG2 cells largely mirrored the biochemical phenotype of cells from the patient. The patient also harboured the homozygous ALG6(F304S) variant, which does not cause CDG but has been reported to be more frequent in PMM2-CDG patients with severe/fatal disease than in those with moderate presentations. WES did not reveal other strong candidate causal genes.

**Conclusions:**

We describe a patient presenting with severe multisystem disease associated with DHDDS deficiency. As retinitis pigmentosa is the only clinical sign in previously reported cases, this report broadens the spectrum of phenotypes associated with this condition.

## Background

**Fig. 1:**
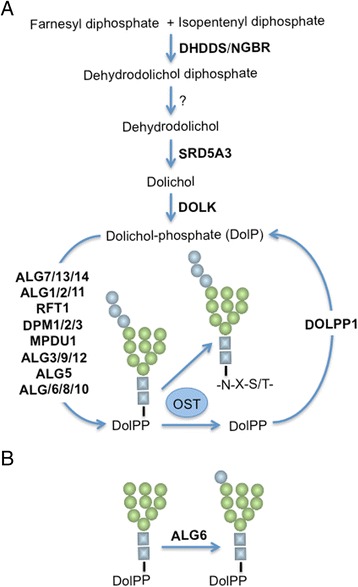
De novo dolichol biosynthesis and the dolichol cycle. **a** Dolichol-phosphate (DolP) is synthesized from farnesyl diphosphate and isopentenyl diphosphate by enzymes encoded by genes indicated in bold capital letters. Enzymes responsible for the dephosphorlylation of dehydrodolichol diphosphate have not been characterised. During the dolichol cycle an oligosaccharide is constructed on DolP by the sequential transfer of sugars (N-acetylglucosamine; *blue squares*, mannose; *green circles*, glucose; *blue circles*) by glycosyltransferases and accessory proteins encoded by genes indicated on the left of the cycle in bold capital letters. The oligosaccharide Glc_3_Man_9_GlcNAc_2_ is transferred onto protein (−N-X-S/T-) by oligosaccharyltranferase (OST) and dolichol-diphosphate (DolPP) is recycled by dolichol diphosphate phosphatase encoded by DOLPP1. **b** The ALG6 gene encodes Dol-P-Glc: Man_9_GlcNAc_2_-PP-dolichol α-1,3 glucosyltransferase

Type I congenital disorders of glycosylation (CDG-I) are rare inborn errors in metabolism A subgroup of CDG-I is caused by mutations in genes involved in the biosynthesis of the dolichol linked oligosaccharide (DLO) precursor required for protein *N*-glycosylation [1]. DLO is synthesized by a series of reactions, known as the dolichol cycle (Fig. 1), in which dolichol phosphate (DolP) is glycosylated with nucleotide sugars and DolP-sugars, to yield the mature DLO, Glc_3_Man_9_GlcNAc_2_-PP-dolichol [2, 3]. The sugar moiety of this donor is transferred from DLO onto nascent polypeptides by oligosaccharyltransferase (OST) while the byproduct of this reaction, dolichol-diphosphate (DolPP), is recycled by a complex process requiring DolPP phosphatase (DOLPP1) [2, 3]. A lack of mature DLO provokes protein hypoglycosylation. In addition to being recycled during protein *N*-glycosylation, DolP is synthesized as one of the end products of the mevalonate pathway (Fig. 1). In the first committed step of dolichol biosynthesis the enzyme cis-prenyltransferase (dehydrodolichol diphosphate synthase: DHDDS) that forms a complex with the Nogo-B receptor (NgBR) elongates a farnesyl diphosphate residue by addition of several isopentenyl moieties derived from isopentenyl diphosphate to yield dehydrodolichol diphosphate [4]. After dephosphorylation of dehydrodolichol diphosphate by poorly characterized enzymes, the resulting dehydrodolichol is reduced by polyprenolreductase (dehydrodolichol reductase or steroid 5α-reductase type 3: SDR5A3) to yield dolichol (Dol) [5] that is subsequently phosphorylated by dolichol kinase (DOLK) to yield DolP (Fig. 1). Dolichol kinase deficiency (DOLK-CDG: MIM610768) presents with hypoglycosylated serum glycoproteins, cardiac myopathy, muscular hypotonia and ichthyosiform dermatitis [6, 7]. Patients with mutations in polyprenolreductase (SDR5A3-CDG: MIM 612379) display hypoglycosylated serum glycoproteins, developmental delay, ataxia, visual impairment (optic nerve atrophy, retinal coloboma, cataract, glaucoma), liver dysfunction, coagulation abnormalities and ichthyosiform dermatitis [5, 8, 9]. NGBR-CDG patients present with scoliosis, neurological impairment, refractory epilepsy, ocular deficits, and visual impairment [10]. By contrast, DHDDS-CDG patients (MIM 613861) have so far not presented with hypoglycosylated serum glycoproteins and clinical signs are limited to retinitis pigmentosa [11, 12]. In the present report we describe biochemical findings in a fatal case of CDG-I in which mutations in DHDDS were found. Two mutations leading to 80 % reduction in DHDDS mRNA were identified. Skin biopsy fibroblasts revealed variable amounts of truncated DLO and *N*-glycans, 35 % residual DHDDS activity, reduced DolP, and down-regulation of DHDDS in HepG2 cells leads to truncated DLO. While whole exome sequencing confirmed the presence of the DHDDS mutations, it did not reveal strong alternative candidate genes involved in protein *N*-glycosylation.

## Methods

### Patient

The boy was the first child of non-consanguineous parents. During pregnancy intra-uterine growth retardation and decreased fetal movements were noted. He was born at 37 weeks of gestation with a weight of 2090 g (−3.25 SD), a length of 42 cm (−4 SD), an occitpiofrontal circumference of 32 cm (−2 SD) and Apgar score 10/10. He had two episodes of severe bradycardia during his first day of life and was transferred to an intensive care unit. At clinical examination, the patient had axial hypotonia, peripheral hypertonia, enlarged liver, micropenis and cryptorchidism. He had a transient increase of serum transaminases, renal failure and developed epilepsy. Liver sonography revealed mild dilatation of the biliary duct. During his short life, the boy made little psychomotor acquisitions, had no eye contact, poor sucking with frequent regurgitations and failure to thrive. At 2 months, the fundus oculi displayed pale papillae and the electroretinogram showed no response to any type of stimulation. Brainstem evoked auditory potentials showed sensorineural deafness with an auditory threshold of 90 dB (right ear) and 100 dB (left ear). The patient died at 8 months during a status epilepticus.

### Reagents

[2-^3^H]mannose (21.5 Ci/mmol), [6-^3^H]glucosamine (37.7 Ci/mmol), [^14^C]-isopentyl diphosphate (56.6 mCi/mmol), GDP-[^14^C]mannose (262 mCi/mmol), UDP-[^14^C]glucose (348 mCi/mmol), UDP-[^3^H]GlcNAc (37 Ci/mmol), ULTIMA GOLD and En^3^Hance were from Perkin Life Sciences, Fr. RPMI 1640 medium, penicillin-streptomycin (PS), fetal calf serum (FCS), OPTIMEM, stealth RNAi, Lipofectamine RNAiMAX and the BCA™ Protein assay kit were from Thermo Scientific, Cergy Pontoise, FR. Peptide N-glycanase, bacitracin, Dowex resins and protease inhibitors were obtained from SIGMA. The tripeptide acetyl-Asn-Tyr-Thr-NH_2_ (Ac-NYT-NH_2_) was purchased from Neosystem (Strasbourg, Fr.). TLC plates were obtained from Merck (Darmstadt, Germany).

### Cell culture

Skin biopsy fibroblasts [13] and HepG2 cells [14] were cultivated as previously described.

### Sequencing

For gene studies, signed informed consent protocols were obtained from the parents. Ethics approval was from the Comité de Protection des Personnes d’Île-de-France 2 (CPP IDF2). Targeted genomic Sanger sequencing of several genes encoding proteins required for DLO biosynthesis [PMM2 (NM_000303), ALG6 (NM_013339), DPM1/2/3 (NM_003859, NM_003863, NM_153741), MPDU1 (NM_004870), ALG2 (NM_033087), ALG7 (NM_001382), ALG1 (NM_019109), ALG13/14 (NM018466, NM_144988), ALG11 (NM_001004127)] and dolichol biosynthesis (DHDDS, NM_020438) was performed on DNA from fibroblasts derived from the patient. Primer sequences are available upon request. DNA from the parents was extracted from whole blood. Informed consent was obtained from the parents. WES libraries were prepared from 3 μg of genomic DNA, which was sheared by ultrasonication using a Covaris S220 Ultrasonicator. The 51 Mb SureSelect Human All Exon kit V5 (Agilent technologies) was used for exome capture, and sequencing of WES libraries was carried out on a HiSeq2500 sequencer (Illumina) [15]. The mean coverage depth was 244.51× ((98.22 > 30×; 99.74 > 15× and 99.93 > 5×). After de-multiplexing, sequences were aligned to the reference human genome hg19 using the Burrows-Wheeler Aligner. Downstream processing was carried out with the Genome Analysis Toolkit (GATK), SAMtools, and Picard, following documented best practices (https://www.broadinstitute.org/gatk/guide/bp_step.php). Variant calls were made with the GATK Unified Genotyper. The annotation process was based on the ensembl database (75), dbsnp (135) EVS (ESP6500SI-V2), 1000 genome (2011 05 21) and EXAC (0.3). Variants were annotated and analysed using the Polyweb software interface designed by the Bioinformatics platform of University Paris Descartes and Imagine Institute [16].

### SiRNA-mediated down-regulation of DHDDS expression in HepG2 cells

HepG2 cells were transiently transfected with 25 pmol siRNA as previously described [14]. Three DHDDS-targetting sense sequences (1; AACAAGUGCAGAUCGCCCAGGACCC, 2; CCAACCCGUUCUGUGGCCAGAGUAU, 3; CCGCUCUCCUCAUCCUGACAUCUUG) and a medium GC non-targetting control sequence were used. Cells were harvested 2, 4 and 6 days after transfection.

### RNA extraction, reverse transcriptase and reverse QPCR

RNA was extracted from fibroblasts or HepG2 cells according to the manufacturer’s instructions (RNeasy Plus Mini Kit, Qiagen). Reverse transcriptase reactions were performed with 1 μg RNA using the Verso cDNA synthesis kit (Thermo Scientific). RT PCR from cDNA exon 3 to 7 of the DHDDS gene (primer sequences: 3SRT cactcacagggcttcaacaagc and 7RRT cggttggtatagaggcacttatc) was performed to confirm the splicing mutation. QPCR was performed using the Absolute Blue QPCR SYBER Green Mix Plus ROX vial (Thermo Scientific) in a Roche Light Cycler 480. Primers for QPCR of DHDDS and the HMBS housekeeping gene were purchased from Qiagen. Primer sequences for the S14 housekeeping gene are as follows: S14 Sense: TCACTCGGAAGAATACCATTTTTG, S14 Reverse; CCGATTTCTGATTCTAACAGGAC.

### Metabolic radiolabelling of cells

Confluent t25 cm^2^ flasks of fibroblasts were metabolically radiolabelled for 30 min in glucose free RPMI 1640 medium supplemented with 0.5 mM glucose and 2 % dialysed foetal calf serum with 100 μCi [2-^3^H]mannose [17]. SiRNA-treated HepG2 cells were radiolabeled for 30 min in 500 μL/well glucose-free RPMI 1640 medium supplemented with 1 mM glucose, 2 mM fucose, 2 % dialyzed FCS and 50 μCi [2-^3^H]mannose [14].

### Characterisation of dolichol linked oligosaccharides and N-glycans

The following experimental procedures are described in detail elsewhere [13]. Briefly, after washing with ice-cold PBS, cells were scraped into 1 volume 100 mM Tris–HCl (pH 7.4) containing 4 mM MgCl_2_. Two volumes of methanol followed by 3 volumes of chloroform were added to the aqueous cell suspension. After vigorous shaking, the CHCl_3_ and MeOH/H_2_O phases were separated by centrifugation and removed. The interface pellet was extracted twice with 3 ml CHCl_3_:CH_3_OH:H_2_O (CMW, 10:10:3). The CHCl_3_ phase and CMW fractions contain DLOs with small and large glycan chains, respectively. Both fractions were dried under vacuum and subjected to acid hydrolysis in 20 mM HCl. After desalting on combined Dowex 1-*X*2 (acetate form)/Dowex 50-*X*2 (H^+^ form) resins the released oligosaccharides were dried under vacuum and, after pooling fractions derived from the two organic phases described above, were examined by thin layer chromatography (TLC). N-linked oligosaccharides (NLOs) were released from the interface protein pellets with PNGase F as previously described [18].

### Dolichol-P-mannose synthase and DHDDS assays

Total HepG2 and fibroblast membranes were prepared as previously described [17]. Dolichol-P-mannose synthase (DPMS) was assayed as reported previously in either the presence or absence of DolP [18]. The same membranes were used to assay DHDDS in either the presence or absence of farnesyl diphosphate (FPP) [19]. For the latter assay, the 50 μL reactions were stopped by the addition of 500 μL CHCl_3_/MeOH (2:1) before transferring the mixtures to 15 mL tubes containing 3.5 mLs CHCl_3_/MeOH (2:1). Two phases were then obtained after addition of 800 μL 4 mM MgCl_2_. The upper phase was removed and discarded whereas the lower phase was washed with 2 × 2 mLs 4 mM MgCl_2_/MeOH (1:1). Radioactivity in the lower phase was then assayed by scintillation counting.

### Detection of endogenous Dol-P

Endogenous Dol-P levels were assayed in sealed ER vesicles as previously described [20]. A sample of sealed ER-vesicles (equivalent to 40 μg protein) was incubated in 50 mM Tris–HCl (pH 7.5) containing: 250 mM sucrose, bacitracin (400 μg/mL), acceptor peptide (35 μM), AMP (4 mM) and CaCl_2_ and MgCl_2_ (5 mM) in a total volume 30 μl. The reaction mixture was incubated for 10 min at 37 °C. GDP-[^14^C]Man (3 μM) was added and the mixture was incubated for 2 min at 37 °C. After solvent extraction, radioactivity was assayed in the organic phase by scintillation counting. The assay was also performed in the absence of the synthetic acceptor peptide as a negative control. Blanks, without sealed ER-vesicles, were made for each condition.

### Thin layer chromatography

Desalted DLOs and NLOs were resolved on silica-coated plastic TLC plates developed for 18 h in a mobile phase of propan-1-ol: acetic acid: water (3:3:2). After drying the plates, radioactive components were visualized by fluorography after spraying with En^3^Hance.

### Protein expression in skin biopsy fibroblasts and HepG2 cells

Proteins were extracted using a lysis buffer containing 20 mM Tris–HCl, 150 mM NaCl, 1 % TX-100, 1 % protease and phosphatase inhibitors, and subjected to SDS-PAGE using NuPage 4–12 % Bis-Tris gels (Fisher-Thermo Scientific). The following primary antibodies were used: anti- ICAM-1 sc-107 (Santa Cruz), anti-PDI (Cell Signaling), anti-ATF-6α (F-7) sc-166659 (Santa Cruz), anti-p-PERK (Thr 981) sc-32577 (Santa Cruz), anti-NgBR IMG-5342-A (Imgenex), anti-Calnexin 610823BD (BD Transduction Laboratories), anti-GRP75 BiP ab-21685 (Abcam), anti-Actin (I-19) sc-1616 (Santa Cruz).

### Complementation of RER2-deficient S. cerevisiae with wild-type and mutant human DHDDS forms

True clone DHDDS (SC321506, NM-205861; 1) was mutated with QuickChange site-directed mutagenesis kit (Stratagene) in K42E and W64X and then subcloned into the yeast p416TEF plasmid [21]. Wt RER2 was obtained by cloning PCR from yeast DNA in plasmid pCR2.1 (TA cloning) and then subcloned into p416TEF. The mutated DHDDS was tested in the RER2-TET-off strain cultivated in the presence of 0.3 μM doxycycline. Yeast strains in which the RER2 (*rer2DR*), ALG14 (*alg14DR*), ALG13 ((*alg13DR*) and ALG7 (*alg7DR*) genes are under the control of the TET repressor were cultivated, alongside a parental strain, in the presence of increasing concentrations of doxycycline for two overnight passages in order to induce gene down regulation. During the second passage, culture growth was measured by turbidometry, and inhibition of growth with respect to that seen in the absence of doxycycline was calculated. Subsequent to cultivation of *alg14DR* and *rer2DR* cells in either the absence or presence of 1.0 and 0.3 μg/mL doxycycline, respectively, as described above, cell extracts [22] were submitted to SDS-PAGE and Western Blot using an antibody directed to carboxypeptidase Y (CPY).

## Results

### Glycosylation profile of plasma glycoproteins

**Fig. 2:**
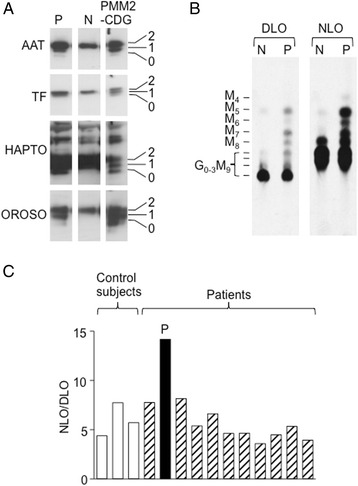
Analysis of serum glycoproteins and N-glycosylation intermediates in fibroblasts. **a** SDS PAGE and Western Blot of serum proteins from a control subject (N), a patient diagnosed with phosphomannomutase 2-deficiencey (PMM2-CDG) and the patient described here (P). Antibodies towards α1-antitrypsin (AAT), transferrin (TF), haptoglobulin (HAPTO) and orosomucoid (OROSO) were used to probe the blots. The numbers to the right of the images indicate glycoforms with 0, 1 and 2 N-glycans. **b** Fibroblasts derived from a control subject (N) and the patient (P) were metabolically radiolabeled with [2-^3^H]mannose and oligosaccharides derived from dolichol linked oligosaccharides (DLO) and glycoproteins (NLO) were resolved by TLC. The abbreviations used are: Man_4-8_GlcNAc_2_; M_4–8_, Glc_0-3_Man_9_GlcNAc_2_; G_0-3_M_9_. **c** Fibroblasts derived from three normal subjects (Ctrls) and five patients diagnosed with the indicated CDG I subtypes were metabolically radiolabeled and, the quantity of radioactivity associated with DLO and pronase-solubilised glycoproteins (N-glycan) was assayed by scintillation counting. The results are expressed as ratio of radioactivity associated with N-glycans/radioactivity associated with DLO

Figure 2a shows Western blotting of the plasma glycoproteins α1-antitrypsin (AAT), transferrin (TF), haptoglobulin (HAPTO) and orosomucoid (OROSO) derived from the patient (P), a patient diagnosed with PMM2-CDG and a normal subject (N). The patient manifests additional AAT, HAPTO and OROSO glycoforms of inferior molecular weight reflecting hypoglycosylation [23].

### Analysis of protein N-glycosylation in patient fibroblasts

Metabolic radiolabelling of skin biopsy fibroblasts with [2-^3^H]mannose revealed that cells from the patient generated elevated levels of truncated DLO possessing oligosaccharides bearing 5-9 residues of mannose as well as fully mannosylated species containing 1–2 residues of glucose (Fig. 2b). N-glycan (NLO) analysis also revealed truncated species bearing 4-8 residues of mannose. The experiment showed in Fig. 2b is representative of several radiolabeling experiments: in some experiments the truncated species were more apparent and in others less so and, more generally, this phenotype gradually became less apparent as a function of cell passage number. As the metabolic radiolabelling procedure is carried out in the presence of low glucose concentrations, which is known to favour the appearance of truncated DLO, we investigated the role of the extracellular glucose concentration on the appearance of truncated DLO in two cell populations derived from separate skin biopsies obtained from the patient. It was noted that both cell populations behaved similarly and that when radiolabeling was performed in the presence of 2 mM glucose (instead of the usual 0.5 mM glucose) truncated DLO were still observed (results not shown). To conclude, oligosaccharide profiles did not point to an obvious block in the dolichol cycle. In addition to truncated DLO, it was noticed that the ratio of the quantity of radioactivity associated with NLO to that associated with DLO was high in cells from the patient compared either to control cells or those from other patients with CDG-I (Fig. 2c).

### Mutations found in DHDDS gene

**Fig. 3:**
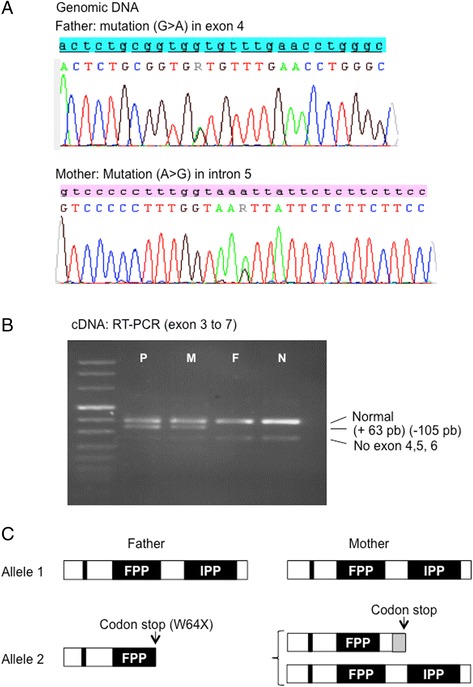
Genomic sequencing and RT-PCR of mRNA transcripts. **a** Genomic DNA sequencing of exon 4 (father) and intron 5 (mother) showing the nonsense mutation (c.192G > A p.W64X) in exon 4, and the splicing mutation (c.441-24A > G) in intron 5. **b** RT PCR from cDNA exon 3 to 7. An alternative transcript without exon 6 exists in all subjects. **c** The domains of the DHDDS protein that contain the isopentenyl diphosphate (IPP) and farnesyl diphosphate (FPP) binding sites are indicated along with the region containing the active site (*vertical bar*). The mutation seen in the father leads to a mRNA containing a premature stop codon which if translated would lead to a severely truncated protein (W64X) missing the IPP binding site. Translation of the mother’s variant mRNA would lead to a severely truncated protein (p.Cys148GlufsX11) containing 11 new amino acids (*grey box*) before the stop codon

Routine targeted Sanger sequencing of several genes encoding proteins required for DLO biosynthesis (PMM2, ALG6, ALG7, ALG13/14, ALG1, ALG2, DPM1/2/3, DOLK MPDU1, and ALG11) did not reveal potential disease causing variants. The patient was found to be homozygous for the c.911 T > C (p.F304S) ALG6 variant (see Fig. 1b) that occurs in about one third of the population and does not cause CDG, but as will be discussed later, has been reported to be associated with severe disease signs in patients whose glycosylation pathway is otherwise compromised. While sequencing genes required for dolichol biosynthesis, two mutations in the DHDDS gene were found in DNA from the patient (Fig. 3a). After sequencing DNA from the parents it was found that the father harboured a nonsense mutation c.192G > A (p.W64X) in exon 4 (Fig. 3a; upper panel), and the mother possessed a splicing mutation (c.441-24A > G) in intron 5 (Fig. 3a; lower panel) that creates a cryptic donor splice site (with score of 0.99 rather than 0.65 for the normal exon 6 donor site) leading to: i) loss of exon 6, ii) a 63 base insertion into intron 5 (Fig. 3b), iii) a premature stop: c.440_543del102ins63 (p.C148EfsX11).

### Complementation of RER2-deficient S. cerevisiae with wild-type and mutant human DHDDS forms

**Fig. 4:**
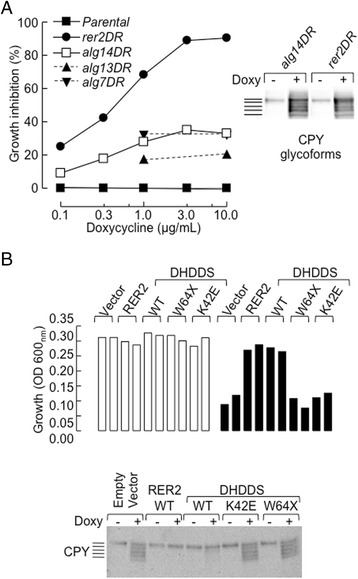
Complementation of rer2-deficient yeast with human DHDDS transcripts*.* **a** (*left panel*). Yeast strains in which the RER2 (*rer2DR*), ALG14 (*alg14DR*), ALG13 (*alg13DR*) and ALG7 (*alg7DR*) genes are under the control of the TET repressor were cultivated, alongside a parental strain, in the indicated concentrations of doxycycline and inhibition of growth with respect to that seen in the absence of doxycycline was calculated. (*Right panel*) Subsequent to cultivation of *alg14DR* and *rer2DR* cells in either the absence or presence of 1.0 and 0.3 μg/mL doxycycline, respectively, as described above, cell extracts were submitted to SDS-PAGE and Western Blot using an antibody directed to carboxypeptidase Y (CPY). The migration positions of different CPY glycoforms are indicated to the left of the image. **b** (*upper panel*) The *rer2DR* strain was transformed with wild type RER2, wild type DHDDS, as well as the DHDDS(W64X) and DHDDS(K42E) variants and the empty vector. Cell growth was measured (OD 600_nm_) in duplicate transformants. (*Lower panel*) CPY glycosylation was examined in one of these duplicate cultures

Both mutations potentially lead to the generation of severely truncated proteins devoid of the isopentenyl pyrophosphate-binding domain (Fig. 3c). A yeast complementation strategy was used to test the functionality of the truncated DHDDS(W64X) protein. In the yeast *S. cerevisiae*, the DHDDS gene is encoded by RER2 and the *rer2Δ* strain is not viable. RER2 function in *S. cerevisiae* can be evaluated using the TET-off system in which the RER2 gene is invalidated upon addition of doxycycline to the culture medium as shown in Fig. 4a. The data shows that down-regulation of RER2 causes striking growth retardation that is more pronounced than that observed when genes required for early steps in DLO biosynthesis (ALG7, ALG13 and ALG14) are down-regulated in the same way. As shown in Fig. 4b, this growth reduction is accompanied by the appearance of hypoglycosylated carboxypeptidase Y (CPY), which is a well-known marker for *N*-glycosylation in *S. cerevisiae*. The functionality of the DHDDS(W64X) variant was tested in the RER2-TET-off strain cultivated in the presence of 0.3 μM doxycycline. Under these conditions, as shown in Fig. 4b, both growth and CPY *N*-glycosylation were restored when the RER2-TET-off strain was transduced with either wild type RER2 or wild type human DHDDS, but not with plasmids containing either DHDDS(W64X) or the DHDDS(K42E) variant that is associated with retinitis pigmentosa.

### Reduced DHDDS gene expression and biological activity in cells from the patient

**Fig. 5:**
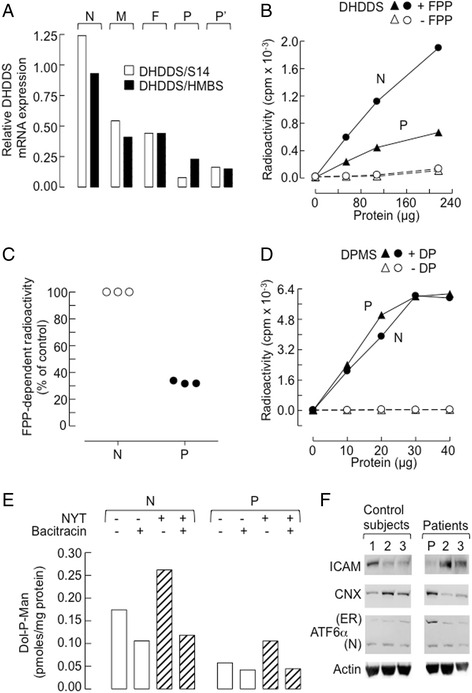
Evaluation of the consequences of DHDDS mutations in fibroblasts. **a** Quantitation of DHDDS cDNA generated from mRNA derived from fibroblasts obtained from two cell populations from the patient (P and P’), a normal subject (N) and whole blood cell extracts from the father (F) and mother (M). QRT-PCR using either ribosomal protein S14 (S14) or hydroxymethylbilane synthase (HMBS) as housekeeping genes was performed. **b** DHDDS activity was assayed using farnesyl diphosphate (FPP) as acceptor. **c** After subtracting background values (− FPP) from the data points, the initial slopes of the curves shown in (**b**) and two other similar experiments were estimated. The values (Slope^P^/Slope^N^) × 100 are plotted (*solid circles*). Slope^N^ is set at 100 % for each experiment (*open circles*). **d** Dol-P-Man synthase (DPMS) activity was measured in the same microsome preparations as used in (**b**) using GDP-[^14^C]mannose and DolP (DP) as acceptor. **e** Measurement of endogenous DolP levels *in* microsomes prepared from fibroblasts derived from the patient (P) and a normal subject (N). Incubations were carried out with GDP-[^14^C]Man in the absence or presence of the glycosylation acceptor peptide (NYT) and bacitracin as indicated. The specific activity of the GDP-[^14^C]Man is used to calculate the pmoles Dol-P-Man recovered from the organic phase after stopping the reactions. **f** Extracts of fibroblasts from three control subjects, the patient (P), and patients diagnosed with ALG12-CDG (Patient 2) and DPM2-CDG (Patient 3) were subjected to SDS-PAGE and Western Blot. The blots were probed with antibodies directed towards intercellular adhesion molecule-1 (ICAM), calnexin (CNX), activating transcription factor 6 alpha (ATF6α) and actin

The DNA sequencing data suggests that cells from the patient should manifest reduced DHDDS expression. The intronic mutation inherited from the mother does not exclude utilization of the normal splice site so some full length DHDDS mRNA is expected in cells from the patient. Full length DHDDS mRNA was quantitated in the skin biopsy fibroblasts from the patient and a normal subject, and, as shown in Fig. 5a, the patient possessed only 20–25 % normal DHDDS mRNA level. DHDDS activity in crude microsomal preparations derived from cells from the patient was found to be 35 % of that found in those of cells from normal subjects (Fig. 5b and c). By contrast, dolichol-P-mannose synthase (DPMS) activity was found to be similar in the two microsome preparations (Fig. 5d).

### Reduced dolichol-phosphate levels in sealed microsomes derived from cells of the patient

The DolP pool available for DLO biosynthesis was estimated in either the absence or presence of a synthetic acceptor peptide (Acetyl-Asn-Tyr-Thr-NH_2_: NYT) that is known to provoke DolP recycling in sealed microsomes [20]. Addition of NYT to sealed microsomes causes DLO to be discharged: yielding glycopeptide and DolPP. DolPP phosphatase (DOLPP1, see Fig. 1) converts DolPP into DolP, which can then be quantitated as Dol-P-[^14^C]Man after addition of GDP-[^14^C]Man and by allowing DPMS action (that occurs at the same levels in microsomes derived from cells of the patient and control subject, Fig. 5d). Bacitracin complexes DolPP and incubations in the absence or presence of this antibiotic allow estimations of both DolPP-, and DolP-dependent Dol-P-[^14^C]Man production. As shown in Fig. 5e, bacitracin inhibits Dol-P-[^14^C]Man production even in the absence of NYT indicating Dol-PP production via the presence of endogenous substrates for OST. Dol-P-[^14^C]Man generation in the presence of bacitracin reveals a pre-existing DolP pool that, in microsomes from the patient, appears to be reduced by ~67 % compared to the control. Finally, it can be seen that microsomes from the patient also demonstrate lower NYT-provoked Dol-P-[^14^C]Man synthesis. The ensemble of these data demonstrates a deficit in the quantity of DolP available for synthesis of Dol-P-[^14^C]Man in the patient’s cells. In order to evaluate the consequences of the reduced DolP levels in the patient’s cells, the glycosylation status of ICAM was examined. Hypoglycosylated ICAM is rapidly degraded and this has been shown to yield low steady state levels of this protein in type I CDG cells [24]. Data presented in Fig. 5f indicate that although the ICAM level is low in the patient’s cells, this level is within the range of ICAM expression in the control cell lines. Protein hypoglycosylation in type I CDG may lead to increases in protein misfolding, ER stress and initiation of the Unfolded Protein Response (UPR) [25]. However, the level of the ER stress marker calnexin (CNX) is within the range displayed by the control cells. Interestingly, there is an inverse correlation between ICAM and CNX expression in both control and patient cells. The reason for the variable ICAM expression even within the control cell population is unknown but could be due to the different growth characteristics or passage number of the different cell lines. ATF46α is a UPR signaling protein [26], and when unfolded proteins accumulate in the ER the expression of full length ATF46α (Fig. 4f, ER; 90 kD) is increased as well as its proteolytic cleavage to generate the 57 kD nuclear from (N) [26]. The ER form of ATF46α is expressed at a higher level in the patient’s cells than in both control cells, and those from other CDG-I patients. However, this increased expression was not accompanied by increased proteolytic cleavage to generate the active nuclear form of the protein (Fig. 5f).

### Knock-down of DHDDS expression in HepG2 cells

**Fig. 6:**
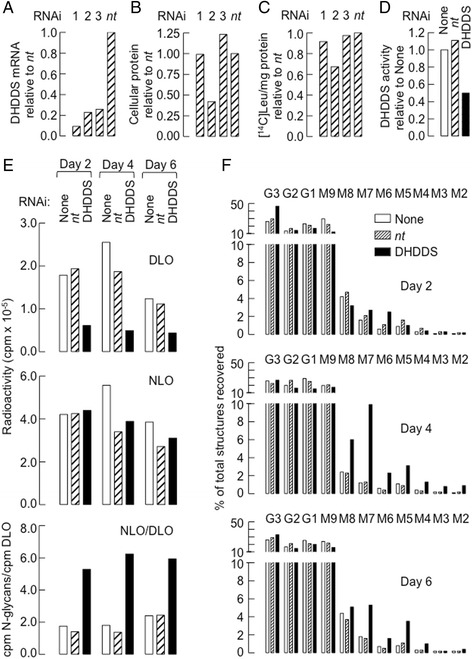
Down-regulation of DHDDS expression in HepG2 cells. HepG2 cells were transfected with three siRNA targeting DHDDS or a control non-targetting siRNA (*nt*) and were harvested 4 days later. **a** Cellular mRNA was extracted and DHDDS cDNA was quantitated by qRT-PCR. **b** Total adherent cell protein was assayed. **c** Cells were radiolabeled with [^14^C]leucine for 30 min, and radioactivity recorded in precipitated cellular proteins was calculated per mg protein and these values were expressed as a percentage of that found in cells transfected with the control non-targetting siRNA. **d** DHDDS activity was measured in the three cell populations. **e** HepG2 cells were mock transfected (None), or transfected with either the control non-targetting siRNA (*nt*) or siRNA(1) targeting DHDDS, and at 2, 4 and 6 days post transfection the cells were metabolically radiolabeled with [2-^3^H]mannose. Radioactivity associated with DLO (*upper panel*) and glycoproteins (NLO: *middle panel*) was measured as described in the legend to Fig. 2c, and the ratio of incorporation of radioactivity into NLO to that into DLO is shown in the lower panel. **f** Oligosaccharides released from DLO were resolved by TLC and visualized by fluorography. Regions corresponding to the migration positions of the indicated oligosaccharides were eluted from the plate and assayed by scintillation counting. The abbreviations are as follows: G3-1; Glc_3_Man_9_GlcNAc_2_, M9-2; Man_9-2_GlcNAc_2_

The data presented above demonstrate the presence of mutations in the DHDDS gene that lead to: 1) reduced DHDDS mRNA levels, 2) reduced DHDDS activity, 3) reduced DolP levels, and 4) small but significant changes in DLO/N-glycan profiles in metobolically radiolabeled fibroblasts derived from the patient. In order to determine whether or not reduced DHDDS expression could lead to the changes in early steps of the N-glycosylation pathway that were noted in the cells from the patient, human hepatocellular carcinoma HepG2 cells were transfected with siRNA duplexes targetting DHDDS. As shown in Fig. 6a, DHDDS mRNA expression was decreased by three DHDDS-targetting duplexes (1-3) compared to the non-targetting duplex (*nt*), and siRNA(1) was the most efficient at reducing DHDDS expression. SiRNA(2) provoked an increase in non adherent cells (not shown), a reduction in protein associated with adherent cells (Fig. 6b), and reduced protein synthesis as measured by [^14^C]leucine incorporation into precipitable cellular proteins (Fig. 6c). Because siRNA(1) provoked more effective DHDDS mRNA down-regulation than siRNA(3) and siRNA(2) manifested toxicity not apparent with the other duplexes, the former duplex was selected for the following experiments. In order to assay DHDDS activity in HepG2 cells that were transfected with siRNA(1), cells were harvested 4 days post transfection and crude microsomal fractions were prepared. Data shown in Fig. 6d indicates a 50 % reduction in DHDDS activity in cells treated with DHDDS-targetting siRNA(1) compared to that found in cells treated with the non-targetting siRNA (*nt*) or sham transfected cells (None). Next, cells were metabolically radiolabeled with [2-^3^H]mannose as described for Fig. 2b. As shown in Fig. 6e, transfection of the cells with the DHDDS-targetting siRNA(1) reduced radiolabel incorporation into DLO (upper panel) without strikingly affecting the quantity of radioactivity associated with N-glycans (middle panel). The resulting high ratio of [2-^3^H]DLO/[2-^3^H]N-glycan (lower panel) in the DHDDS-down-regulated cells is similar that seen with the fibroblasts derived from the patient (Fig. 2c). TLC examination of the DLO structures generated under the different conditions demonstrates the presence of various truncated DLO associated with DHDDS down-regulation at days 4 and 6 post-transfection (Fig. 6f). Finally, down-regulation of DHDDS in HepG2 cells did not lead to changes in the expression of ICAM, calnexin and ATF46α (results not shown). To summarize, as was noted in fibroblasts from the patient, DHDDS down-regulation in HepG2 cells provokes the appearance of truncated DLO and an elevated [^3^H]DLO/[^3^H]N-glycan ratio.

### Whole exome sequencing

The clinical phenotype of the patient reported here resembles that of other CDG-I patients and contrasts with the retinitis pigmentosa phenotype of the first reported DHDDS-deficient patients. This different phenotype could be explained by the presence of a mutation in another gene that regulates N-glycosylation, or the association of DHDDS mutations with mutations in genes that regulate N-glycosylation. Sanger sequencing revealed the patient to be homozygous for the c.911 T > C (p.F304S) ALG6 variant (see Fig. 1b) that does not cause CDG, but is associated with severe disease signs in patients who have mutations in other genes of the N-glycosylation pathway. In order to look for other potentially disease causing or disease modifying variants, whole exome capture and next-generation sequencing (WES) on an Illumina platform was performed on DNA from fibroblasts of the patient. After filtering out variants occurring at > 1 % in human population data banks (the c.911 T > C (p.F304S) ALG6 variant was detected but filtered out because it occurs at a frequency of 20-30 %), and selecting those associated with glycosylation or dolichol metabolism pathways, two genes were found with two variants: DHDDS and TNKS1 (Tables 1 and 2). TNKS1 (Tankyrase, TRF1-Interacting Ankyrin-Related ADP-Ribose Polymerase) functions include NAD^+^-dependent ADP-ribosyltransferase activity*.* Poly-ADP-ribosyltransferases are involved in various processes such as Wnt signaling, telomere length maintenance and vesicle trafficking [27]. In certain situations, the chromosomal location of the TNKS1 gene (8p23.1) is linked to a form of monogenic diabetes [28]. Despite being lean and in other respects normal, TNKS1-deficient mice display hyperphagia and altered fatty acid metabolism without liver steatosis [28]. WES also identified several heterozygous variants in genes of the N-glycosylation pathway (Table 2) including ALG8, ALG9, DDOST, MPDU1, ALG6, and STT3A. Even though single mutations in these genes do not cause CDG, the possibility that reduced activity/expression of the proteins that they encode could amplify the phenotype caused by reduced DHDDS expression cannot be excluded. The possibility of further CDG gene variants hiding in an intron or poorly covered region during WES cannot be excluded, but it was found that for the 26 genes presently known to underlie CDG, WES coverage was at least 30×.

**Table 1:** Whole exome sequence filtering

SNVs^a^	Variations	Genes
All variants	9061	6301
Unknown or known variants <1 % (dbSNP132/1 K genome/EVS ExAC) and in-house database filtering (929)^b^	785	861
Glycosylation genes Dolichol related genes	19 3	19 3
Gene(s) with two mutated alleles	2	2

^a^ *SNVs* single nucleotide variants

^b^dbSNP132: http://www.ncbi.nlm.nih.gov/SNP/

1 K genome; 1000 genomes (http://www.1000genomes.org/), EVS; Exome Variant Server (http://evs.gs.washington.edu/EVS/), ExAC; http://exac.broadinstitute.org/, in-house database filtering; variant excluded if already seen at the homozygous state in 929 exomes performed in the platform

**Table 2:** .Glycosylation or dolichol related genes with mutated alleles

Gene	SNP^a^	Type of mutation^b^	Frequency data if already described^c^
DHDDS	c.192G > A (p.W64X)	Stop	-
	c.441-24A > G (p.C148EfsX11)	Intron (splice)	ExAC: 0.00001663
TNKS	c.1945G > A (p.D649N)	Missense (Polyphen pathogen, SIFT benign)	-
	c.899-16362A > T	Intron/ncRNA	-
ALG8	c.1068C > G (p.P356=)	Exon synonymous	-
ALG9	c.-37-77G > A	Intron	-
DDOST	c.679A > G (p.I227V)	Missense (Polyphen and SIFT benign)	ExAC: 0.00004444
MPDU1	c.393C > T, p.V131 = (rs79286384)	Exon synonymous	Av. he: 0.004 ExAC: 0.0007853
ALG6	c.987 + 43 T > C (rs181709997)	Intron	Av. he: 0.003 ExAC: 0.003458
STT3A	c.88 + 131 T > C (rs191172467)	Intron	Av. he: 0.001+/−0.024 ExAC: no data

^a^Genomic positions are on UCSC genome Browser (hg19)

^b^Polyphen : http://genetics.bwh.harvard.edu/pph/, SIFT: http://sift.jcvi.org/

^c^Av. he: average heterozygosity (source dbSNP) or allele frequency ExAC

## Discussion

This case is of considerable interest because other patients so far described with mutations in the DHDDS gene present with a mild clinical picture restricted to retinitis pigmentosa [11, 12, 29]. The present patient showed a fatal clinical syndrome accompanied with hypoglycosylated serum glycoproteins and truncated DLO species, pointing to CDG-I. Two mutations in DHDDS leading to 20–25 % DHDDS mRNA expression and 35 % residual DHDDS activity were found. In accordance with these data microsomes derived from the patient’s cells revealed low levels of DolP. As our studies progressed, and the passage number of the fibroblasts increased, we noted a gradual disappearance of the glycosylation phenotype. Although it is not clear why this should occur, it is possible that as the cells become less metabolically active their glycosylation requirements are reduced and there is less strain on the dolichol cycle. The disappearance of the glycosylation phenotype, and the fatal outcome in this case, made it no longer feasible to try to normalize either the abnormal DLO profile or [2-^3^H]mannose incorporation into DLO by transfecting cells from the patient with wild-type hDHDDS. In an alternative approach to investigate the origin of the biochemical phenotype, DHDDS expression was down regulated in HepG2 cells. To date, there have been no attempts to knockdown DHDDS expression in mice or mammalian cells in vivo. It was noted that whereas DHDDS-down regulation in HepG2 cells provoked an 80–90 % reduction in DHDDS RNA, only a 50 % reduction in DHDDS activity could be detected. This result was surprising and raises the possibility of an alternative mechanism for dolichol biosynthesis that is relatively more important in HepG2 cells than in fibroblasts. Alternative dolichol biosynthetic routes postulated previously [30, 31] remain controversial [32]. Nevertheless, such a route might explain the shorter dolichol chain lengths that have been observed in NGBR- and DHDDS-deficient patients [10, 33]. Here we demonstrate that siRNA-mediated knockdown of DHDDS did not reduce the incorporation of [2-^3^H]mannose into glycoproteins, but did reduce strikingly the incorporation of [2-^3^H]mannose into DLO, and provoked a transient increase in the proportion of truncated DLO species (Man_6-8_GlcNAc_2_-PP-dolichol). One explanation for the occurrence of truncated DLO in DHDDS-deficient cells with reduced DolP is that the synthesis of Dol-P-Man and Dol-P-Glc are more adversely affected than Dol-PP-GlcNAc. This would lead to a DLO profile similar to that seen in MPDU1-CDG where the ability of these two precursors to contribute to DLO elongation is compromised [34]. Alternatively, the slightly shorter dolichol chains that have been shown to occur in previously described DHDDS- and NGBR-deficient patients may also hinder Dol-P-Man and Dol-P-Glc utilization. The mechanism underlying the paradoxical apparent increase in incorporation of radioactivity into N-glycans with respect to that incorporated into DLO is also not clear. One explanation might be that the reduced DolP pool size could lead to a higher specific activity of the radioactive DPM and DLO pools compared to control cells. Accordingly, assuming that protein synthesis rates are similar and that the DLO pools are not limiting in control and DHDDS-deficient cells then a higher incorporation of radioactivity into N-glycans might be expected. An alternative hypothesis for the inability of DHDDS down regulation to inhibit the incorporation of radioactivity into N-glycans despite reducing radioactivity associated with DLO is that the DLO pool required for protein glycosylation is a sub pool of total DLO, and that this sub pool is less dependent upon DHDDS than the remaining DLO. To summarise, in both cells from the patient and DHDDS-deficient HepG2 cells, changes in the incorporation of [2-^3^H]mannose into DLO and N-glycans along with changes in the DLO profiles were detected. Therefore it is concluded that the biochemical phenotype of cells from the patient is compatible with reduced DHDDS expression.

The previously described patients with mutations in the DHDDS gene that present only with retinitis pigmentosa [11, 12, 29], do not present with serum glycoprotein hypoglycosylation. In these patients serum and urine dolichols are abnormal [33]. Serum and urine samples from our patient were not available. The DHDDS ortholog, RER2, is an essential gene in yeast, and DOLK-CDG, SRD5A3-CDG and NGBR-CDG patients, who have mutations in other genes required for the *de novo* biosynthesis of dolichol, present with moderate to severe multisystemic manifestations [5, 10, 35]. Several explanations are possible for the differences in these DHDDS-CDG phenotypes.

First, the K42E mutation, associated with isolated retinitis pigmentosa, is perhaps not particularly damaging. Functional glycosylation studies and DHDDS assays have not been performed in fibroblasts from these patients, so no comparison can be made with the findings in cells from the present patient. Nevertheless, we report here that wild-type hDHDDS, but not hDHDDS(K42E), is able to normalise both impaired growth and carboxypeptidase Y hypoglycosylation in RER2-deficient *S. cerevisiae*. Interestingly, we present data indicating that doxycycline-induced knockdown of other essential yeast glycosylation genes (ALG7, ALG13 and ALG14) has much less impact on *S. cerevisiae* growth than down-regulation of RER2. This observation suggests that the RER2-dependent dolichol pool is required for important cellular functions other than protein N-glycosylation, or that RER2 itself has a role in yeast homeostasis independent of its role in dolichol production. Although the hDHDDS(K42E) variant is unable to complement growth or N-glycosylation defects in *S. cerevisiae,* its ability to support N-glycosylation in mammalian/human cells maybe quite different due to the fact that efficient dolichol biosynthesis by the hDHDDS-encoded protein requires the hNGBR-encoded protein [4, 10, 36]: a complex between hDHDDS(K42E) and the yeast NgBR ortholog (nus1) may not be as productive as that comprising both human proteins. More recently, however, it has been demonstrated that membranes derived from *nus1Δrer2Δ*yeast complemented with hDHDDS(K42E) and wild-type hNGBR display about 30 % the activity of those derived from the same strain complemented with wild-type hDHDDS and hNgBR [10]. Therefore hDHDDS(K42E) does not support efficient DHDDS activity.

Second, the reduced DHDDS activity caused by the K42E variant may not be too serious for cells (apart from those in the retina). However, reduced expression of the DHDDS protein may be far more serious. DHDDS knockdown in fertilized zebrafish eggs caused primarily retinal photoreceptor degeneration, but, depending on the degree of knockdown, provoked other phenotypic changes that suggest roles for DHDDS are not restricted to retinal photoreceptor maintenance in this organism. Accordingly, reduced DHDDS expression may be more damaging than normal expression of the inactive hDHDDS(K42E) variant, potentially suggesting that DHDDS has important functions that are independent of its DHDDS activity. Nevertheless, an understanding of the impact of DHDDS knockdown on mammalian physiology will have to await the generation of mice models. The DHDDS protein forms a complex with the NgBR to form the fully active dehydrodolichol diphosphate synthase activity. NgBR also forms a complex of unknown function with the Nogo-B protein, and a complex with the Niemann-Pick C2 protein (NPC2) that is known to regulate cholesterol metabolism. NGBR knockout is embryonically lethal in mice and NGBR-deficient cells, derived from either patients with mutations in NGBR or mouse embryonic fibroblasts derived from conditional NGBR^−/−^ mice, display 10-17 % residual DHDDS activity, reduced ICAM expression and about 50 % reduction in [2-^3^H]mannose incorporation into glycoproteins [10]. Accordingly, one of the consequences of reduced DHDDS protein expression, that is independent of reduced enzyme activity, might be an excess of “free” NgBR that could deregulate cholesterol metabolism or pathways that depend on the Nogo-B protein.

Third, Sanger sequencing revealed that the patient was homozygous for the ALG6(F304S) variant, which is more frequent in PMM2-CDG patients with severe disease than in those with moderate symptoms [37]. The ALG6(F304S) variant is not as efficient at complementing ALG6-deficient yeast as the wild type allele [38], but is not thought to cause CDG because it occurs in 27–33 % of the population, and 4–6 % of the population are homozygous for this variant [37, 39, 40]. DLO analyses in fibroblasts derived from the patient did reveal accumulations of Man_9_GlcNAc_2_-PP-dolichol above control levels (Fig. 2b), but other DLO species accumulated to a greater extent, and this DLO profile is not indicative of ALG6-CDG. WES of our patient revealed two variants in the tankyrase 1 (TNKS1) gene along with heterozygous variants in other genes (ALG6, ALG8, ALG9, DDOST, MPDU1, and STT3A) required for N-glycosylation. A role for these variants in the fatal outcome of this disease cannot be excluded.

## Conclusions

We describe a patient presenting with severe multisystem disease associated with DHDDS deficiency. As retinitis pigmentosa is the only clinical sign in previously reported cases of DHDDS deficiency our data broaden its phenotypic spectrum. This case adds to those in which the ALG6(F304S) variant is associated with a severe/fatal disease presentation.

## Abbreviations

CDG, congenital disorders of glycosylation; DHDDS, dehydrodolichol diphosphate synthase; DLO, dolichol linked oligosaccharide; DolP, dolichol phosphate; DolPP, dolichol diphosphate; NgBR, Nogo-B receptor; OST, oligosaccharyltransferase; siRNA, short interfering RNA.
